# Retention of a Swallowed Dental Tool in the Small Bowel: Unusual Lodgment and Prolonged Conservative Management

**DOI:** 10.1155/crid/5586308

**Published:** 2024-12-16

**Authors:** Ken Chapman Kigozi, Nevis Agirembabazi, Andrew Marvin Kanyike, Racheal Nalunkuma, Rachael Mukisa Nakandi, Simon Peter Nsingo, Robinson Ssebuufu

**Affiliations:** ^1^Department of Dentistry, Mengo Hospital, Kampala, Uganda; ^2^School of Dentistry, Uganda Christian University, Mukono, Uganda

**Keywords:** asymptomatic retention, conservative management, dental tool, foreign body, small bowel

## Abstract

Accidental ingestion of foreign bodies during dental procedures is relatively common, with the potential for serious complications, including intestinal perforations and severe pulmonary disorders. Our case report describes the prolonged, asymptomatic retention of a swallowed hex driver in the small bowel of a 38-year-old male, managed conservatively over an extended period. The patient, with a significant medical history of depression, was undergoing a dental procedure involving implant-supported restorations when the incident occurred. Initial x-rays confirmed the presence of the hex driver in the gastrointestinal tract. Despite its stationary position for over a week, it passed naturally without requiring invasive intervention. This case highlights the importance of individualized patient care and deferring recommendations for intervention in certain instances. We recommend a more individualized approach to managing ingested foreign bodies in dental settings, emphasizing the need for tailored strategies based on the patient's specific circumstances and clinical stability.

## 1. Introduction

Accidental swallowing of foreign bodies (FBs) is common among people of all ages, especially during dental procedures [1, 2]. The high-risk patients are the elderly, children, and adults with mental illness and anxiety [3]. Endodontic instruments, implant devices, teeth, and prostheses are the most commonly swallowed FBs in dental clinics [4]. Previous studies have shown that ingestion occurs more frequently than aspiration, mostly during prosthodontic treatment [5, 6].

FBs ingested mainly pass through the gastrointestinal tract (GIT) without issue, with only 10%–20% requiring endoscopic intervention [7]. In a previous study, more than 90% of patients who ingested FBs and 50% of aspirated FBs were asymptomatic [8]. While most cases remain asymptomatic, critical complications can occur if an FB is swallowed or aspirated during dental treatment. Ingested FBs can result in ulcerations, impactions, intestinal perforations, and sepsis [9]. Similarly, aspiration through the airway can cause a range of severe pulmonary disorders [9, 10].

The management strategies for these incidents vary depending on factors like the patient's age, overall health condition, the location and properties of the object, the time elapsed since ingestion, and the technical capabilities of the endoscopist and facility [7]. Generally, conservative outpatient management is appropriate for clinically stable asymptomatic patients when an ingested object goes beyond the esophagus, as the object will often pass spontaneously [7].

Our case report details the incident of a 38-year-old male who swallowed a hex driver during a dental procedure. This case illustrates the prolonged asymptomatic retention of a swallowed dental tool in the small bowel, managed conservatively over an extended period. The case highlights the importance of individualized patient care, with the need to delay unnecessary invasive interventions.

## 2. Case Presentation

**Figure 1:**
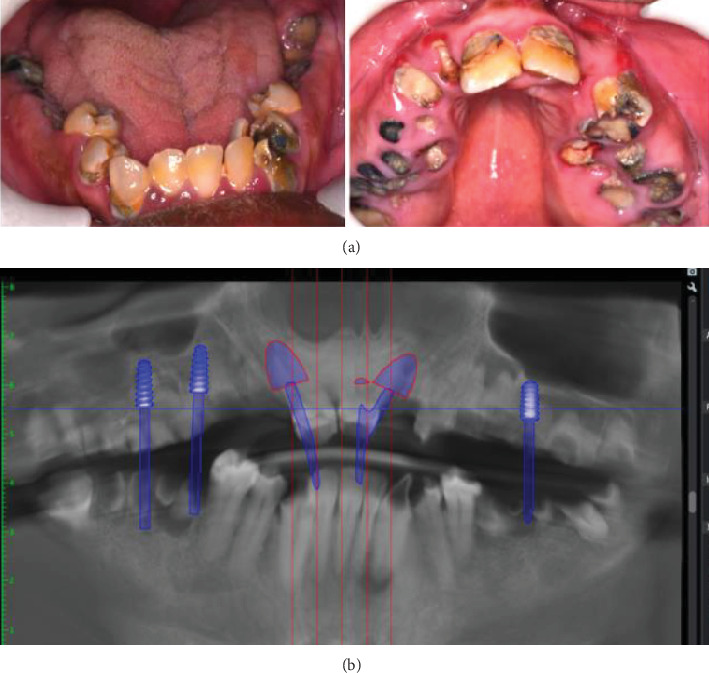
(a) Multiple dental caries with many broken teeth in both upper and lower jaw. (b) Upper jaw with limited bone height below the maxillary sinuses.

A 38-year-old male with a significant medical history of being treated for depression with monthly risperidone injections first presented to our hospital on February 13, 2020. At this initial visit, a comprehensive dental examination revealed widespread dental caries and numerous broken-down teeth (Figure 1(a)). After evaluating various treatment options, which included the extraction of nonrestorable teeth followed by either removable partial/complete dentures or implant-supported restorations, a decision was made in consultation with his caretakers and implant specialist to proceed with fixed implant-supported restorations under the “All-on-X” protocol. The upper jaw x-ray revealed limited bone height below the maxillary sinuses, requiring a sinus lift procedure for one of the maxillary implants while planning the All-on-X restorations (Figure 1(b)).

Preparatory treatments began in early April 2022, with the extraction of all remaining teeth and roots and the fabrication of removable upper and lower complete dentures. The previously fabricated dentures were attached to the newly placed implants, creating fixed, nonremovable provisional upper and lower restorations. The patient reported satisfactory function with these provisional restorations, though some of his relatives thought the teeth were excessively large, which was adjusted in the final restoration.

Two additional upper implants were placed on August 10, 2022, one of which involved a sinus bone grafting procedure. The patient continued using the fixed provisional restorations while awaiting the osseointegration of his implants and the construction of his final restorations. The final upper restoration was placed in October 2023, and he continued using the adjusted lower provisional restoration until the final lower restoration was placed on February 23, 2024.

**Figure 2:**
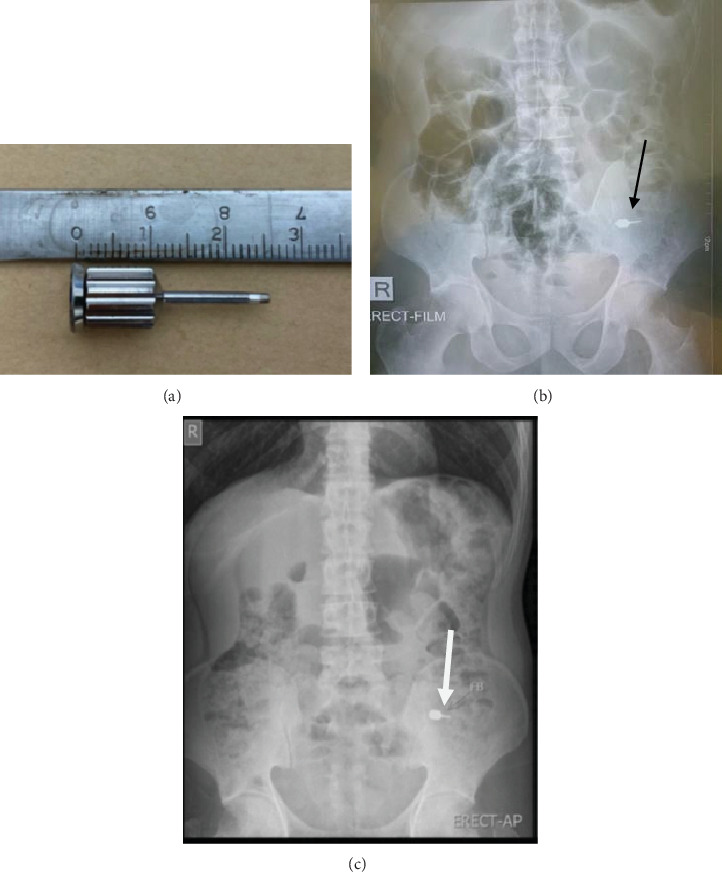
(a) Hex driver similar to the one swallowed about 2.5 cm long. (b) X-ray taken immediately after the swallowing incident showing hex driver in the small bowel (black arrow). (c) X-ray is taken 6 days after the incident with the hex driver in the same position (white arrow).

**Figure 3:**
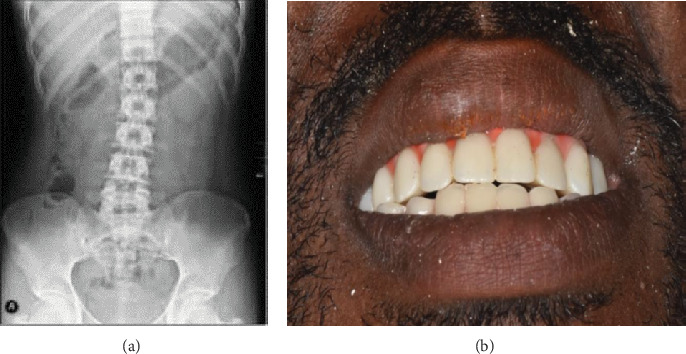
(a) An x-ray was done 2 weeks after the swallowing incident. (b) The patient after complete upper and lower jaw restorations.

During the fitting session for the final lower all-on-four restoration on the same day, he swallowed a hex driver about 2.5 cm long (Figure 2(a)). An x-ray taken immediately showed the hex driver within the small bowel (Figure 2(b)). A follow-up x-ray taken 6 days later showed no movement of the hex driver (Figure 2(c)). However, he reported no discomfort or other symptoms, and regular bowel movements continued. Upon consultation with the surgical and endoscopy teams, retrieving the instrument according to protocol was suggested. However, consensus was reached to pursue a more conservative approach. He was advised to eat sweet yellow bananas and steamed cabbage to move the instrument. A final x-ray done another week later confirmed the hex driver had passed naturally from his body (Figure 3(a)).

The patient achieved functional and aesthetically pleasing dental restorations and was complimented on the appearance of his new teeth. Regular reviews have been planned for ongoing monitoring (Figure 3(b)).

## 3. Discussion

Foreign object ingestion during dental procedures can be a significant challenge, ranging from harmless to severe complications [4]. Although most FBs ingested in dental settings do not cause symptoms, it is crucial not to overlook the potential for severe outcomes such as intestinal perforations or sepsis. A study reported the perforation rate of the GIT with sharp FBs is 35% [11]. FBs in the GIT should be evaluated based on their type, size, shape, and location [4].

Imaging studies offer insights into FBs' location, shape, and characteristics. Fortunately, most foreign objects contain radiopaque materials that can be easily identified on x-rays [7]. Radiological follow-up is necessary to track the FB in cases of ingestion. As long as the patient remains asymptomatic, scans can be taken weekly to evaluate the progress of small blunt objects [12]. When an FB is swallowed, the size, sharpness, and shape of the ingested FB should be considered. The risk of injury increases when the object's size is more than 5 cm or has a pointed shape [13, 14]. If a blunt and short object is swallowed and enters the stomach, conservative management is the choice, as most objects spontaneously pass through the digestive system within 4–6 days [7]. However, if the object is spherical and has a diameter of more than 2.5 cm (or smaller in pediatric patients), it is less likely to pass through the pylorus. However, if the object remains in one exact location for more than 1 week or is retained for more than 2–4 weeks (depending on its composition), it should be removed endoscopically [7, 15].

Our case illustrates a scenario where an FB (a hex driver) of about 2.5 cm remained in the small bowel for more than a week in the same anatomical position without symptomatic complications. This finding is consistent with the fact that most ingested objects pass through the body without any problem; however, the fact that the object remained in the same anatomical position for more than a week is problematic, and guidelines suggest intervention in such cases. In instances where it gets stuck in the upper GI, a minimally invasive laparoscopic approach is considered safe for removal of the ingested FB and preferred to open surgery [16]. However, in this case, the object unusually got stuck in the left iliac hemiabdominal region. This area usually does not have narrowing or strictures to hold an object. This happens in specific areas such as the ileocecal region, appendix, sigmoid colon, or rectum. However, any part of the intestine can be affected, particularly in patients with bowel disorders like adhesions or inflammatory bowel disease [2]. Studies suggest that if an object gets stuck in the small intestine beyond the reach of a standard upper endoscope, enteroscopy (e.g., push, balloon-assisted, and laparoscopically assisted) can help access and remove the stuck object [17].

Although there was no prior GI disease or surgery history, the prolonged retention of the object created suspicion and anticipation of obstruction. However, in contrast to the evidence of taking a surgical approach, we considered conservative management with a prolonged observation period due to the absence of symptoms and complications. The extended duration before the natural expulsion of the FB, in this case, with the FB lasting more than 1 week in the same anatomical place, prompts always reconsidering timelines to intervention beyond the recommendations and taking an individualized approach. This case demonstrates that with continuous monitoring and the absence of acute symptoms, extending conservative management may be a viable option. This is especially pertinent given that sharp objects have a higher rate of causing perforations, at about 35%, whereas blunt objects like the hex driver, in this case, are less likely to cause such complications [4].

The case also highlights and calls for embracing preventive measures to reduce the risk of FB ingestion during dental procedures. Difficult handling of small, slippery instruments in a confined working area, excessive or unexpected patient movements during treatment, limited mouth opening, and unexpected breakage or detachment of poor-quality instruments or their components are significant contributing factors to the ingestion of FBs in dentistry [1]. Rubber dams, dental floss tied to instruments, and thorough patient history assessments are recommended to minimize risks [2, 3]. Thorough patient history assessments are also essential to identify conditions such as anxiety or limited mouth opening that may require additional precautions. In light of this case, these measures should be reinforced and also integrated into protocols and guidelines that account for the specific type of FB, the patient's overall health, and real-time imaging findings to inform tailored management strategies.

## 4. Conclusion

The case is constructive in understanding the boundaries of conservative management for ingested FBs in dental settings. It suggests that even extended durations of FB retention in the GIT can be managed conservatively with proper monitoring. This case underscores the need for individualized care plans based on specific clinical scenarios. It supports the push for more adaptive guidelines to accommodate a broader range of patient responses and outcomes.

## Data Availability

The data that support the findings of this study are available on request from the corresponding author. The data are not publicly available due to privacy or ethical restrictions.
